# Data on synthesis and characterization of chitosan nanoparticles for *in vivo* delivery of siRNA-*Npr3*: Targeting NPR-C expression in the heart

**DOI:** 10.1016/j.dib.2016.05.074

**Published:** 2016-06-03

**Authors:** Balaji Venkatesan, Anusha Tumala, Vimala Subramanian, Elangovan Vellaichamy

**Affiliations:** Department of Biochemistry, University of Madras, Guindy campus, Chennai 600025, India

**Keywords:** Chitosan nanoparticles, Gene silencing, Biocompatibility, Hemocompatibility

## Abstract

This data article contains the data related to the research article ‘Transient silencing of *Npr3* gene expression improved the circulatory levels of atrial natriuretic peptides and attenuated β-adrenoceptor activation-induced cardiac hypertrophic growth in experimental rats’ (Venkatesan et al., 2016 [1]). The siRNA-*Npr3* loaded chitosan nanoparticles were synthesized using ionotropic gelation method, where the positive charge of the chitosan interacts with the negative charge of STPP and siRNA-*Npr3.* The physicochemical properties of the synthesized siRNA-*Npr3* loaded chitosan nanoparticles were studied by dynamic light scattering, FE-SEM and HR-TEM analysis. In addition, the loading efficiency and stability of the nanoparticles were also studied. Further, the gene silencing efficacy, hemocompatibility and biocompatibility were studied using Wistar rats (*in vivo*), isolated red blood cells and H9c2 cardiomyoblast cells, respectively.

**Specifications Table**

**Table:** 

Subject area	Biology
More specific subject area	Nanotechnology, Molecular biology.
Type of data	Figure.
How data was acquired	FE-SEM, HR-TEM, DLS, Zeta potential, agarose gel electrophoresis, hemocompatibility assay, MTT assay, RT-PCR and Western blotting.
Data format	Raw and analyzed.
Experimental factors	Synthesis of siRNA-*Npr3* loaded chitosan nanoparticles by ionotropic gelation.
Experimental features	Hypertrophied H9c2 cells and hypertrophied rats were treated with different concentration of target 1 and 2 siRNA-*Npr3* loaded chitosan nanoparticles to validate its gene silencing efficacy.
Data source location	NA.
Data accessibility	Data are available within this article.

**Value of the data**

• This data describes the synthesis and characterization of the siRNA-*Npr3* loaded chitosan nanoparticles for in vitro and in vivo applications.
• This data validated the biocompatibility, hemocompatibility and gene silencing efficacy of siRNA-*Npr3* loaded chitosan nanoparticles in *in vitro* and *in vivo* model system.
• This method of siRNA-*Npr3* loaded chitosan nanoparticles can be utilized as a drug delivery vehicle for *in vitro* and *in vivo* applications.

## 1 Data

The data provided here displays the synthesis, characterization, biocompatibility and hemocompatibility of siRNA-*Npr3* loaded nanoparticles. Further, the gene silencing efficacy of the synthesized siRNA-*Npr3* nanoparticles was demonstrated in the H9c2 cells *in vitro* and in rat hearts *in vivo.*

## 2 Experimental design, materials and methods

### 2.1 Materials

Chitosan (75–85% of deacetylation, low molecular weight), *Npr3* specific siRNA – Target 1: 5′-GUUUGCUAAUGGCCUUCUA[dT][dT]-3′ and 5′ UAGAAGGCCAUUAGCAAAC[dT][dT]-3′ (CAT# SASI_Rn01_00055729), Target 2: 5′-GACUAUGCUUUCUUCAACA[dT][dT]-3′ and 5′-UGUUGAAGAAAGCAUAGUC[dT][dT]-3′ (CAT# SASI_Rn02_00260355) and sodium tripolyphosphate were procured from Sigma-Aldrich, USA. Dulbecco׳s Modified Eagle׳s Medium (DMEM), Trypsin-EDTA, fetal bovine serum (FBS) and antibiotic antimycotic solution were purchased from HiMedia, India. cDNA conversion kit was procured from Thermo scientific, USA. Red dye PCR master mix was purchased from Merck Millipore, German. Gene specific primers were purchased from Eurofins Scientific, Luxembourg. Primary antibody for NPR-C and HRP labeled secondary antibody were purchased from Santa Cruz biotechnology, USA.

### 2.2 Synthesis and characterization of siRNA-*Npr3* loaded chitosan nanoparticles

The siRNA-*Npr3* loaded chitosan nanoparticles were synthesized by mixing chitosan solution (1 mg/ml chitosan in 0.2 M sodium acetate buffer, pH 4.5) to a mixture containing STPP solution (2.5 mg/ml) and siRNA-*Npr3* at 5:1 weight ratio of chitosan to STPP and 50:1 N:P ratio of chitosan and siRNA-*Npr3*. The contents were mixed and vortexed for 1 min on a vortex mixer and kept undisturbed for 30 min [2]. The synthesized nanoparticles were purified by centrifuging at 31,000*g* for 20 min. Further, the nanoparticles were washed with ultrapure DNase/RNase free water and centrifugation was repeated.

**Fig. 1:**
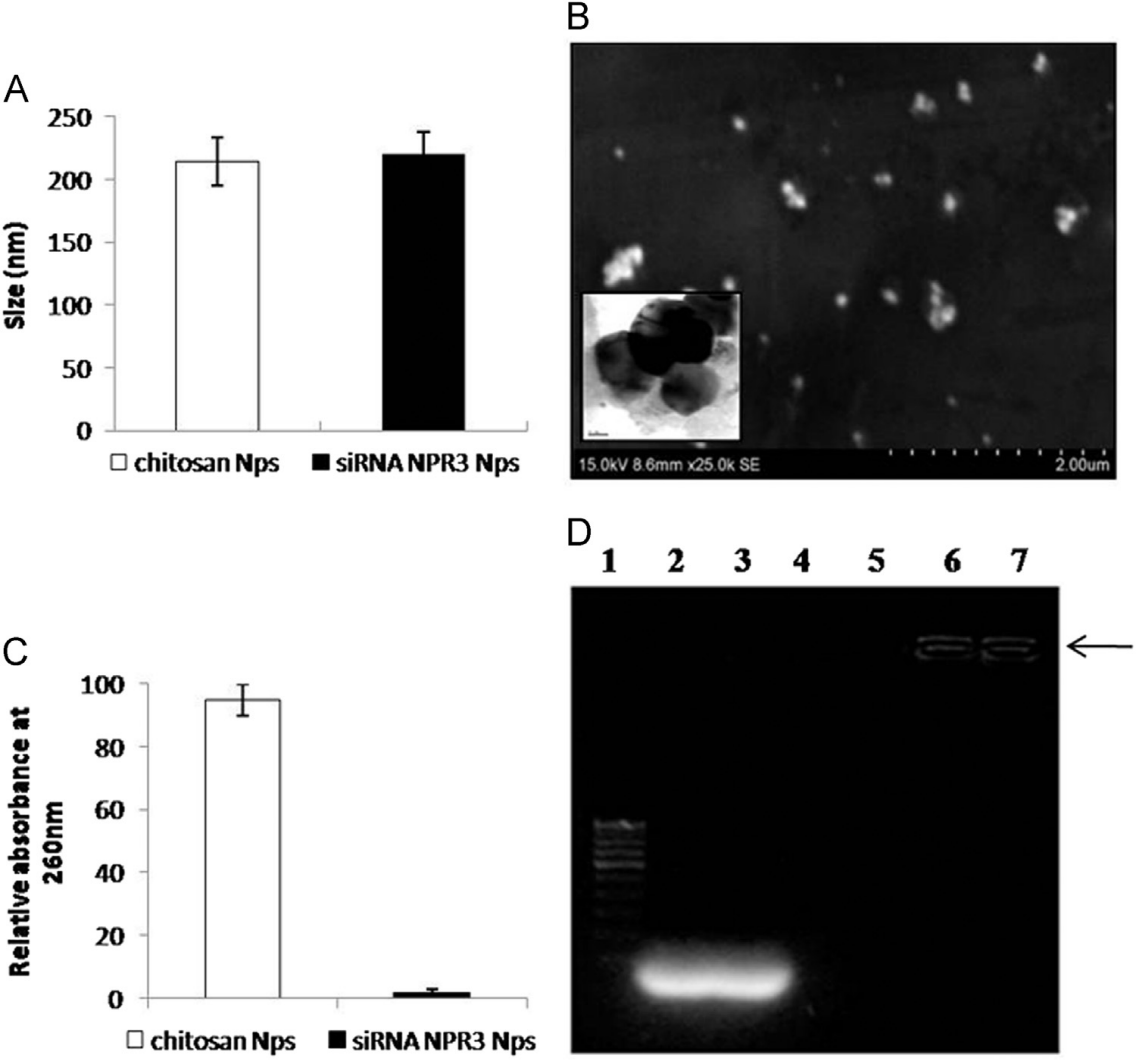
Synthesis, characterization and stability of siRNA-*Npr3* loaded chitosan nanoparticles: A) Dynamic light scattering analysis, B) FE-SEM analysis; insert in B) HR-TEM analysis, C) Loading efficiency of the nanoparticles D) Representative gel retardation assay of siRNA-*Npr3* loaded chitosan nanoparticles where, Lane-1: 100 bp DNA ladder, Lane-2: nacked siRNA-*Npr3* target 1, Lane-3: nacked siRNA-*Npr3* target 2, Lane-4 and 5: chitosan nanoparticles, Lane-6: siRNA-*Npr3* loaded chitosan nanoparticles (target 1), Lane-7: siRNA-*Npr3* loaded chitosan nanoparticles (target 2).

Fig. 1A shows the particle size analysis (DLS – Malvern Nano ZS, UK) of synthesized nanoparticles, which revealed that the hydrodynamic size of the siRNA-*Npr3* nanoparticles were in the range of 220±17.5 nm. From the zeta potential analysis, the nanoparticles were found to possess a surface charge of +16.2±1.2 mV. It is evidenced from the FE-SEM – Fig. 1B (Hitachi SU6600, Germany) and HR-TEM – insert in Fig. 1B (FEI TECNAI G2) analysis of the nanoparticles that the synthesized particles were of fairly spherical in shape and evenly distributed.

### 2.3 Quantification of siRNA-*Npr3* loading efficiency

The loading efficiency of the siRNA-*Npr3* in the siRNA-*Npr3* loaded chitosan nanoparticles were analyzed using UV–Vis spectrophotometer (Shimadzu UV 150-02, Japan) by comparing the A_260_ of the supernatant solution obtained after the synthesis of siRNA-*Npr3* loaded chitosan nanoparticles and the naked siRNA-*Npr3* [3]. The loading efficiency was calculated by using the following formula: Loading efficiency=A_260_ nm of siRNA present in the supernatant/A_260_ nm of total amount of siRNA added for nanoparticles preparation*100 and the siRNA-*Npr3* loading efficiency were observed to 100% (Fig. 1C).

### 2.4 Gel retardation assay

The stability and interaction strength between the chitosan and siRNA-*Npr3* in the nanoparticles was carried out by gel retardation assay. Briefly, equal concentration of naked siRNA-*Npr3*, chitosan nanoparticles and siRNA-*Npr3* loaded chitosan nanoparticles were loaded on different wells of 4% agarose gel for analyzing the stability of the nanoparticles [3]. The gel retardation assay showed that the nanoparticles loaded with siRNA-*Npr3* gets retarded in the well as evidenced by the retarded movement of the nanoparticles, while the naked siRNA-*Npr3* freely resolved in the agarose gel (Fig. 1D).

### 2.5 Biocompatibility of siRNA-*Npr3* loaded chitosan nanoparticles

**Fig. 2:**
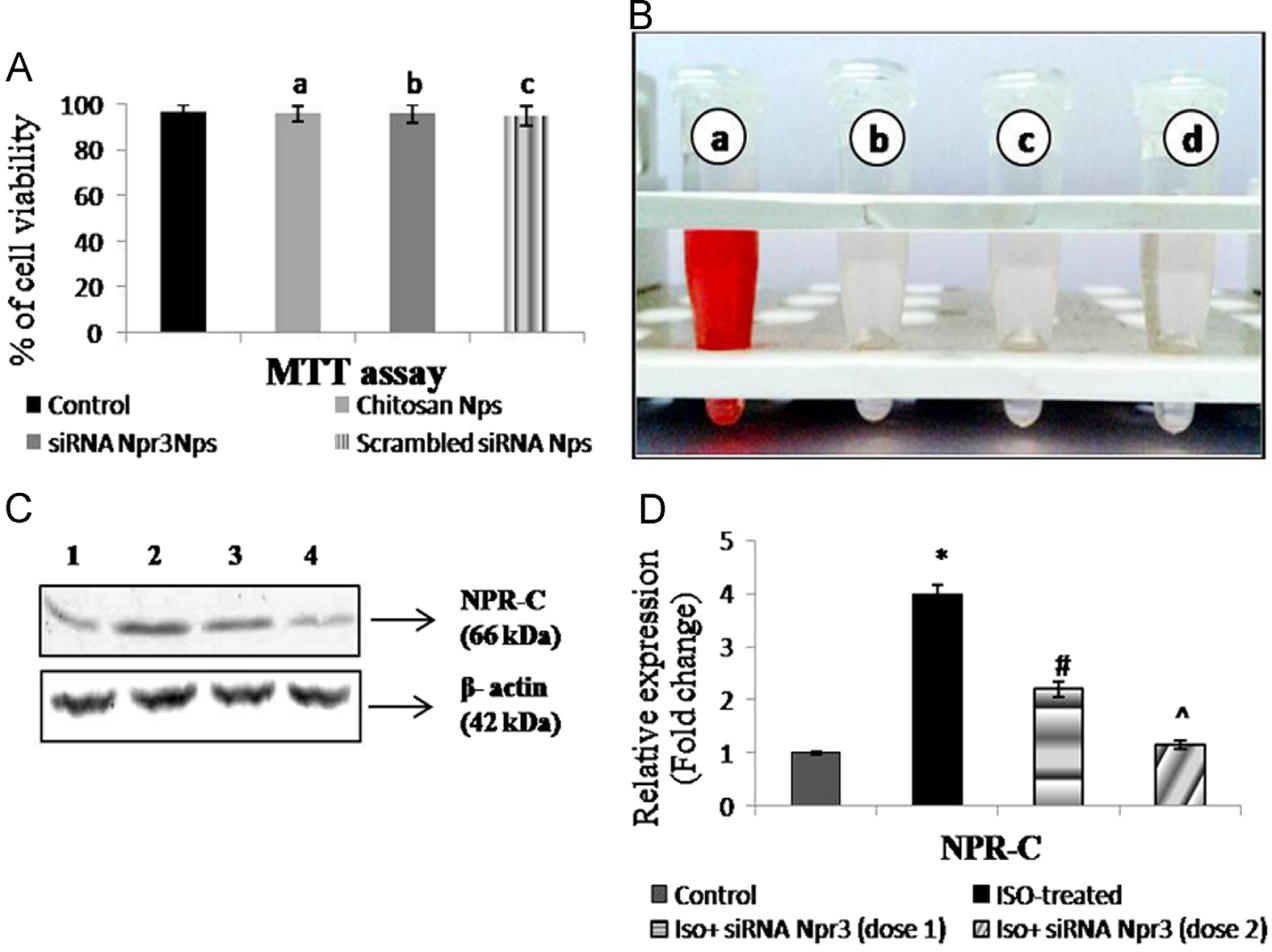
Biocompatibility, hemocompatibility and targeting efficacy of siRNA-*Npr3* loaded chitosan nanoparticles, *in vivo***:** A) cytotoxicity of chitosan nanoparticles on H9c2 cells by MTT assay. Data are represented as mean±S.E.M. (n=3), where, a – untreated vs chitosan nanoparticles (Non-significant), b – untreated vs siRNA-*Npr3* nanoparticles (Non-significant), c – untreated vs scrambled siRNA nanoparticles (Non-significant), B) Representative image of hemocompatibility of the chitosan nanoparticles on erythrocytes where, (a) treatment with 1% triton X 100 (positive control), (b) treatment with chitosan nanoparticles, (c) treatment with siRNA-*Npr3* loaded chitosan nanoparticles and (d) treatment with scrambled siRNA nanoparticles. C) Representative immunoblot analysis of NPR-C protein expression on treatment with different dose of siRNA-*Npr3*, where lane 1 – Control, 2 – ISO-treated for 7 days, 3 – ISO+siRNA-*Npr3* nanoparticles (2.5 µg/kg body weight), 4 – ISO+siRNA-*Npr3* nanoparticles (5 µg/kg body weight). D) Densitometric analysis of NPR-C immunoblot. Data are represented as mean±S.E.M. (n=6 rats). *-*P*<0.01 – Control vs ISO-treated, ^#^-*P*<0.05 – ISO-treated vs ISO+siRNA-*Npr3* (dose 1), ^-*P*<0.01 – ISO-treated Vs ISO+siRNA-*Npr3* (dose 2).

To assess the biocompatibility of the nanoparticles, MTT assay was carried out on H9c2 cell line. Briefly, 5000 cells/well were seeded in 96 well plate and maintained in 10% FBS containing DMEM for 24 h. After 24 h, the cells were treated with either chitosan nanoparticles or siRNA-*Npr3* loaded chitosan nanoparticles or chitosan scrambled siRNA nanoparticles in serum free media for 48 h. Later, the media was removed and 10 µl of MTT solution (5 mg/ml) was added to each well and incubated for 4 h at dark. After the reaction, the MTT was removed and 100 µl of DMSO was added to all the wells. The A_570_ was read by microplate reader [4]. Fig. 2A shows the results of the biocompatibility assay, where none of the nanoparticles (chitosan, siRNA-*Npr3* loaded chitosan nanoparticles, and chitosan scrambled nanoparticles) tested exhibits cytotoxicity against H9c2 cells.

### 2.6 Hemolytic activity

The hemolytic activity of the nanoparticles was tested on isolated rat erythrocytes [5]. Briefly, the whole blood was processed and erythrocytes were collected in a vial. 5% v/v erythrocytes in PBS were distributed to each tube and the volume of the tube was made up to 1 ml with nanoparticles samples (chitosan nanoparticles or siRNA-*Npr3* loaded chitosan nanoparticles or chitosan scrambled siRNA nanoparticles) and PBS. Erythrocytes treated with 1% triton X 100 served as the positive control and erythrocytes in PBS served as negative control. The reaction mixture was incubated for 1 h at 37 °C in shaking condition. Then the tubes were centrifuged at 190*g* and the absorbance of the supernatant was measured at 540 nm. The percentage of hemolysis=[(A_540_ nm in siRNA-*Npr3* loaded chitosan nanoparticles supernatant solution – A_540_ nm in PBS)/(A_540_ in 1% Triton X-100 – A_540_ in PBS)]×100. Fig. 2B, shows the results of the hemolytic activity assay. The nanoparticles (150 µg/ml) were tested individually on rat erythrocytes and found to be exhibit least toxicity as per ASTM standard which can be considered as compatible [6].

### 2.7 Validation the gene silencing efficacy of siRNA-*Npr3* loaded chitosan nanoparticles

The *in vivo* gene silencing efficacy and effective dosage fixation of siRNA-*Npr3*loaded chitosan nanoparticles were performed by intramyocardial injection of the nanoparticles. Briefly, 2.5 or 5 µg of siRNA-*Npr3* containing nanoparticles/kg body weight was administered to the hypertrophied Wistar rats. At the end of the experiment, the heart tissue was harvested and processed as described earlier [1]. Fig. 2C and D shows the Western blotting and densitometric analysis of NPR-C protein expression on the control and experimental group of rat hearts, where a 4 fold increase in the NPR-C protein expression was observed in the isoproterenol treated rat hearts, and treatment with 2.5 or 5 µg of siRNA-*Npr3* decreased the expression of NPR-C by 45% and 70% respectively.

**Fig. 3:**
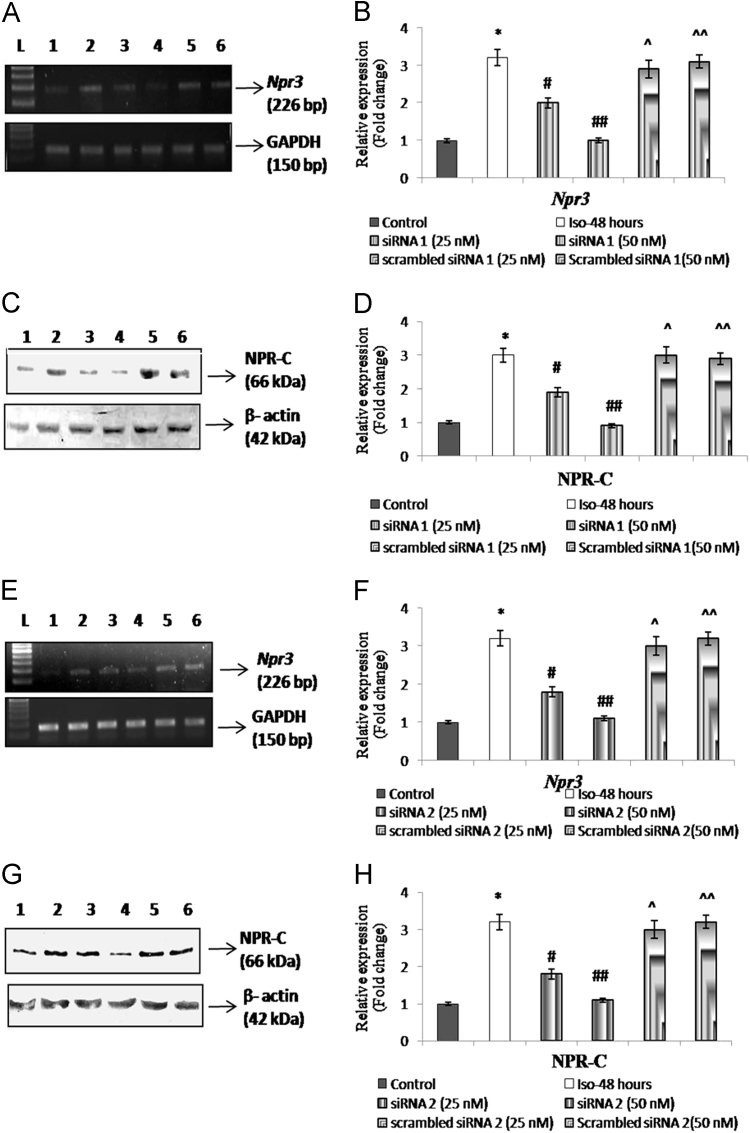
Validation of siRNA-*Npr3* loaded chitosan nanoparticles on H9c2 cells *in vitro***:** A) and B) Representative RT-PCR and densitometry of *Npr3* gene expression, respectively. C) and D) Representative immunoblot and densitometry of NPR-C protein expression, respectively, where L-100 bp DNA ladder, Lane 1 – control, 2 – ISO-treated, 3 – ISO+siRNA target 1(25 nM), 4 – ISO+siRNA target 1(50 nM), 5 – ISO+scrambled siRNA target 1(25 nM), 6 – ISO+scrambled siRNA target 1(50 nM). Data are represented as mean±S.E.M. (n=3). *-*P*<0.01 – Control vs ISO-treated, ^#^-*P*<0.05 – ISO-treated vs ISO+siRNA target 1(25 nM), ^##^-*P*<0.01 – ISO-treated vs ISO+siRNA target 1(50 nM), ^-Non-significant – ISO-treated vs ISO+scrambled siRNA target 1(25 nM), ^^-Non-significant – ISO-treated vs ISO+scrambled siRNA target 1 (50 nM). E) and F) Representative RT-PCR and densitometry of *Npr3* gene expression, respectively. G) and H) Representative immunoblot and densitometry of NPR-C protein expression, respectively, where L-100 bp DNA ladder, Lane 1 – control, 2 – ISO-treated, 3 – ISO+siRNA target 2(25 nM), 4 – ISO+siRNA target 2(50 nM), 5 – ISO+scrambled siRNA target 2(25 nM), 6 – ISO+scrambled siRNA target 2(50 nM). Data are represented as mean±S.E.M. (n=3). *-*P*<0.01 – Control vs ISO-treated, ^#^-*P*<0.05 – ISO-treated vs ISO+siRNA target 2(25 nM), ^##^-*P*<0.01 – ISO-treated vs ISO+siRNA target 2(50 nM), ^-Non-significant – ISO-treated vs ISO+scrambled siRNA target 2(25 nM), ^^-Non-significant – ISO-treated vs ISO+scrambled siRNA target 2 (50 nM).

To validate the gene silencing efficacy of the siRNA-*Npr3* loaded chitosan nanoparticles, H9c2 cells were seeded on 6 well plates (0.15×10^6^ cells/well) and treated as follows: 1) control cells-no treatment; 2) ISO treated-ISO (10 µM); 3) ISO+25 nM siRNA-*Npr3* (target 1); 4) ISO+50 nM siRNA-*Npr3* (target 1); 5) ISO+25 nM scrambled siRNA (target 1); 6) ISO+50 nM scrambled siRNA (target 1); 7) ISO+25 nM siRNA-*Npr3* (target 2); 8) ISO+50 nM siRNA-*Npr3* (target 2); 9) ISO+25 nM scrambled siRNA (target 2); 10) ISO+50 nM scrambled siRNA (target 2). At the end of 48 h of treatment, NPR-C expression was analyzed by RT-PCR and Western blotting analysis. The RT-PCR and Western blotting analysis showed a significantly (3-fold) increased *Npr3* gene expression on treatment with ISO. Upon co-treatment with siRNA target 1 (25 nM) and target 2 (25 nM), the level of *Npr3* gene expression was found to be decreased by 1.5-fold, respectively (Fig. 3A–H).
