# Supplementary material for: Data on synthesis and characterization of chitosan nanoparticles for in vivo delivery of siRNA-Npr3: Targeting NPR-C expression in the heart
Source: Data Brief. 2016 Jun 3;8:441–7. doi: 10.1016/j.dib.2016.05.074 (PMC4910299; doi:10.1016/j.dib.2016.05.074)
Supplement: Supplementary file 1 — Supplementary material [file mmc1.docx]

**Conflict of Interest**

The authors- Balaji Venkatesan, Anusha Tumala, Vimala Subramanian and Elangovan Vellaichamy has no conflict of Interest.
