# Supplementary figures and images for: Data on synthesis and characterization of chitosan nanoparticles for in vivo delivery of siRNA-Npr3: Targeting NPR-C expression in the heart
Source: Data Brief. 2016 Jun 3;8:441–7. doi: 10.1016/j.dib.2016.05.074 (PMC4910299; doi:10.1016/j.dib.2016.05.074)

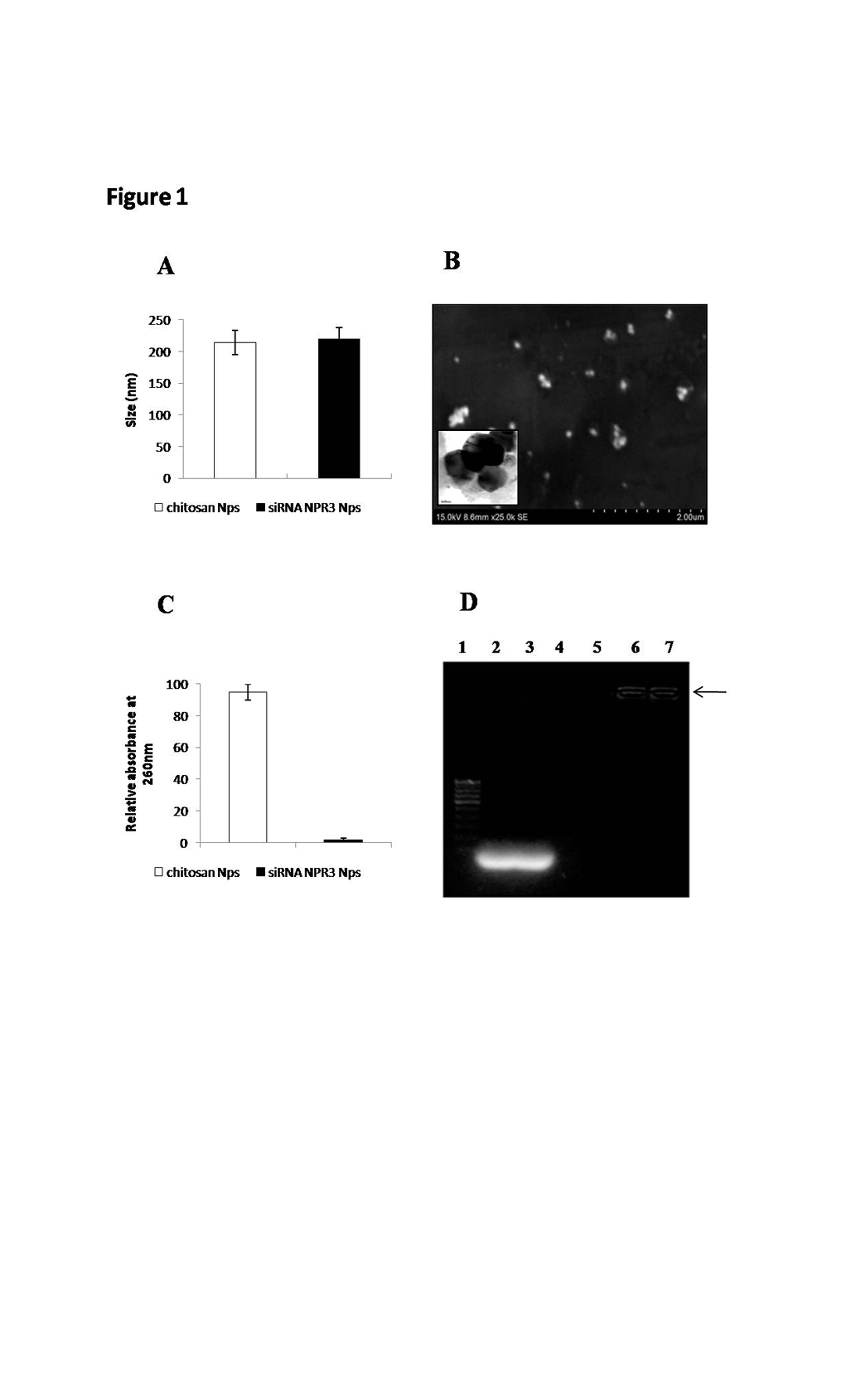

Supplement: Supplementary file 2 — Supplementary material [file mmc2.zip › Figures/Figure 1.jpg]

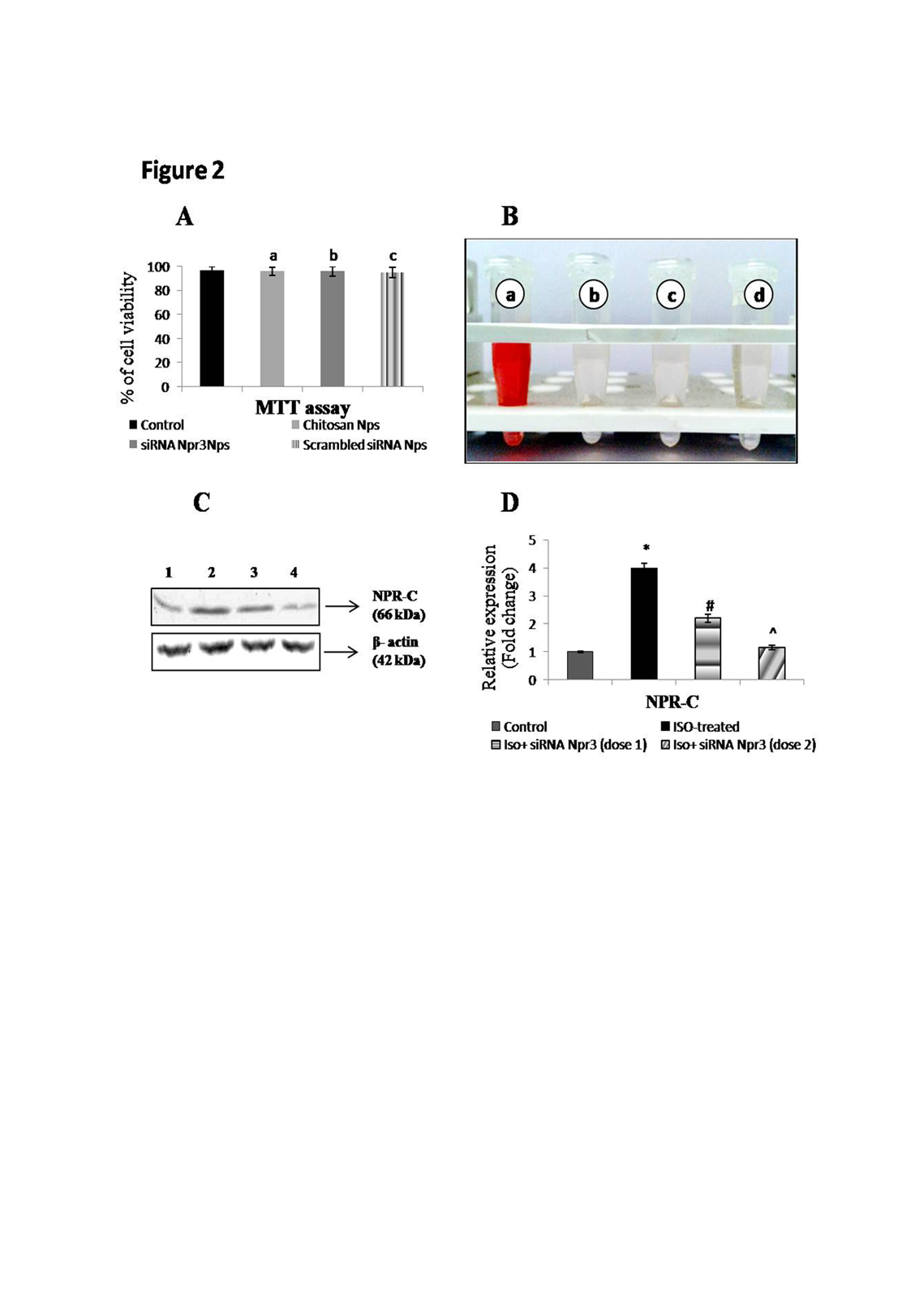

Supplement: Supplementary file 2 — Supplementary material [file mmc2.zip › Figures/Figure 2.jpg]

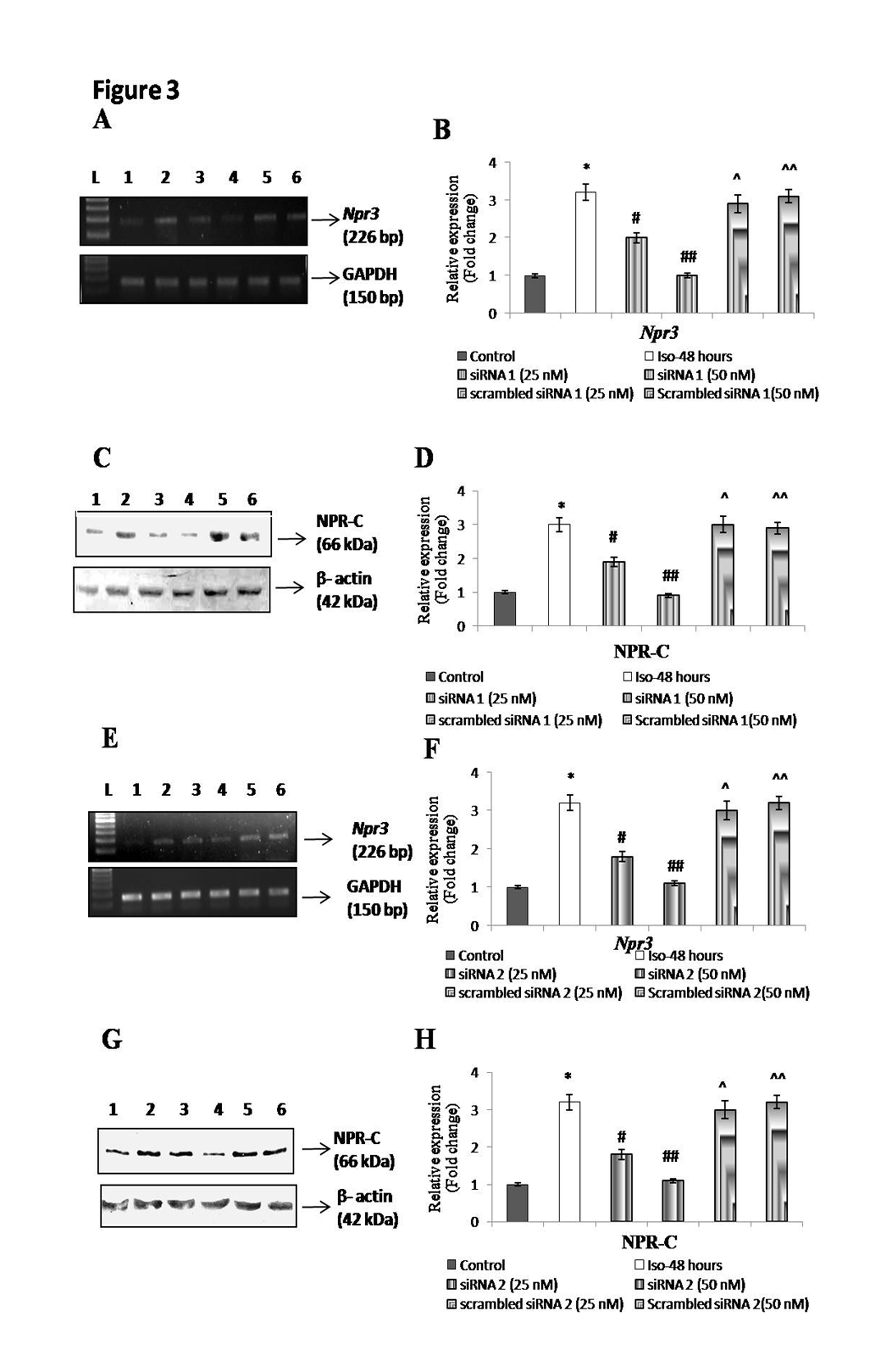

Supplement: Supplementary file 2 — Supplementary material [file mmc2.zip › Figures/Figure 3.jpg]
